# Association of Physicians’ Self-Compassion with Work Engagement, Exhaustion, and Professional Life Satisfaction

**DOI:** 10.3390/medsci7020029

**Published:** 2019-02-12

**Authors:** Oksana Babenko, Amber D. Mosewich, Ann Lee, Sudha Koppula

**Affiliations:** 1 Department of Family Medicine, Faculty of Medicine & Dentistry, University of Alberta, AB T6G2T4, Canada; ann.lee@ualberta.ca (A.L.); skoppula@ualberta.ca (S.K.); 2 Faculty of Kinesiology, Sport, and Recreation, University of Alberta, AB T6G2H9, Canada; amber.mosewich@ualberta.ca

**Keywords:** physicians, self-compassion, professional wellbeing

## Abstract

Self-compassion has shown promise as an adaptive resource for coping with uncertainties and challenges. This study examined the relationship between self-compassion and professional wellbeing (work engagement, exhaustion, and professional life satisfaction) of physicians, who frequently face uncertainties and challenges in their clinical practice. Fifty-seven practicing physicians in Canada participated in the study. Overall, 65% of the participants were female; 47% were in the early-career stage; 49% were family medicine (FM) physicians, with the rest being non-FM specialists. It was hypothesized that (a) self-compassionate physicians would experience greater work engagement and less exhaustion from work than physicians reporting lower self-compassion and (b) self-compassionate physicians would experience greater professional life satisfaction through their greater work engagement and less exhaustion than physicians reporting lower self-compassion. Sequential regression analyses were performed. The results confirmed the hypothesized associations, indicating that self-compassionate physicians experienced more positive work engagement, felt less emotionally, physically, and cognitively exhausted due to work demands, and were more satisfied with their professional life than physicians who exhibited less compassion toward themselves in uncertain and challenging times. Future studies are needed to determine optimal ways to support practicing physicians and medical trainees in becoming more self-compassionate for their enhanced wellbeing and, ultimately, for the provision of effective patient care.

## 1. Introduction

Despite numerous rewards, healthcare practice is inherently stressful, requiring a mindset and a skillset to respond effectively to challenges and in particular to those challenges that bear uncertainty (e.g., clinical ambiguity, close calls, and therapeutic setbacks) [1,2]. While patients heavily rely on physician knowledge in reducing clinical uncertainty when establishing a diagnosis, determining a prognosis, and formulating a treatment plan, patients also rely on physician skill in responding to uncertainty [1,3,4]. The means by which healthcare providers respond to uncertainties and arising challenges are critical and impact systems, healthcare providers, and the patients they serve.

Empirical research indicates that healthcare providers’ poor coping with uncertainties is linked to suboptimal clinical decision-making (e.g., premature diagnosis, increased testing, and referrals) and decreased healthcare provider wellbeing (e.g., stress, anxiety) [2,4]. Concomitantly, published literature reports high levels of professional dissatisfaction and burnout, characterized by work disengagement and exhaustion [5,6], among physicians and other healthcare providers globally [7]. In Canada, the economic cost of early retirement and reduction in clinical hours of practicing physicians due to physician burnout is estimated at $213.1 million [8]. Taken together, these findings are disconcerting, as the provision of effective and safe patient care rests on healthcare provider wellbeing and satisfaction with professional life [9].

As an adaptive way of coping, responding to uncertainty and challenges *self-compassionately* [10] has shown promise across various domains and aspects of life (e.g., personal relationships, counseling, school, sport, and military), helping individuals maintain a balanced perspective in the face of uncertainty, protecting them from negative automatic responses, and enhancing their functioning and wellbeing [10,11]. Self-compassion encompasses three components: self-kindness versus self-judgment, a sense of common humanity versus isolation, and mindfulness versus over-identification [10]. Self-kindness involves being understanding and accepting towards oneself in times of uncertainty and challenges, as opposed to being overly self-critical and highly judgmental. Common humanity involves recognizing that one’s experiences are not isolating and that others also encounter uncertainties and challenges. Mindfulness involves experiencing one’s thoughts and feelings as they are, rather than suppressing, avoiding, or excessively reacting to them.

Extensive empirical research supports associations between self-compassion and a variety of beneficial outcomes, including enhanced task engagement, performance, and wellbeing [10,11]. For example, in a randomized intervention study with teachers, Roeser and colleagues showed that increased self-compassion mediated reductions in teachers’ stress and burnout at the 3-month follow-up [12]. In the context of healthcare practice, a recent systematic review of empirical studies has shown mindfulness (one of the components of self-compassion) to be associated with reductions in burnout, distress, anxiety, depression, and stress [13]. A number of other authors suggested that explicitly training medical learners and practitioners to respond to uncertainties and challenges with a self-compassionate approach will enhance their professional wellbeing and, ultimately, the quality of patient care [1,11,14].

**Figure 1:**
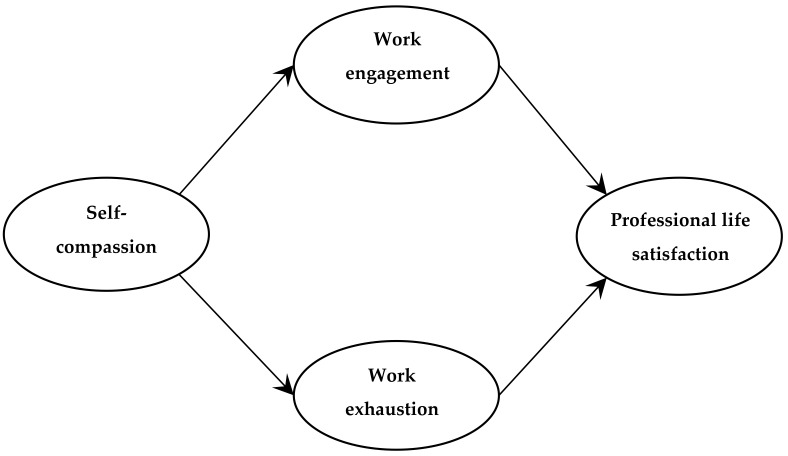
Hypothesized relationships of self-compassion with work engagement, work exhaustion, and professional life satisfaction.

The objective of the present study was to investigate the association of self-compassion with professional wellbeing of practicing physicians, specifically in relation to their work engagement, exhaustion (emotional, physical, and cognitive), and professional life satisfaction. In this study, the degree of work engagement was framed in light of work disengagement, which is defined as distancing oneself from one’s work in general [5,6]. Work exhaustion is defined as a consequence of chronic, intensive physical, emotional, and cognitive strain (i.e., a long-term consequence of prolonged exposure to certain work demands) [5,6]. Both disengagement and exhaustion are aspects of work-related burnout [5,6]. Life satisfaction, and by extension professional life satisfaction, is defined as a conscious cognitive judgment of one’s (professional) life on the basis of the individual’s own unique set of criteria [15]. In the present study, the following hypotheses were tested (Figure 1):

- Hypothesis (1): Self-compassionate physicians experience greater work engagement and less exhaustion from work than physicians reporting lower self-compassion.
- Hypothesis (2): Self-compassionate physicians experience greater professional life satisfaction through their greater work engagement and less exhaustion than physicians reporting lower self-compassion.

## 2. Materials and Methods

This study is part of a larger research project investigating personal and contextual factors in the learning and wellbeing of medical students and practicing physicians. The findings on student and physician motivation and lifelong learning have been reported elsewhere [16,17,18,19]. In the present study, we examined the association of physicians’ self-compassion with work engagement, exhaustion, and professional life satisfaction.

### 2.1. Study Design and Participants

This was a survey study using an online questionnaire composed of validated measures. The questionnaire link was circulated through professional mailing lists and announcements at professional events (e.g., national conferences). Ethics approval (#Pro00066510) was obtained from the Research Ethics Board at the University of Alberta, Canada, prior to data collection. Participation in the study was voluntary and participants could choose to omit a question. No remuneration was provided to the participants. Fifty-seven practicing physicians in Canada completed the survey: 65% were female; 47% were in the early-career stage (≤10 years in practice); 49% were family medicine (FM) physicians, with the rest being non-FM specialists (e.g., internal medicine, pediatrics, surgery). 

### 2.2. Measures

#### 2.2.1. Self-Compassion

The 12-item self-compassion scale–short form (SCS-SF) [20] was used to measure self-compassion in practicing physicians. Using a five-point Likert-type scale (1—almost never; 5—almost always), participants were asked to indicate how often they behaved in a certain way with respect to their work. Sample items are: “When something painful happens, I try to take a balanced view of the situation” and “I try to see my failings as part of the human condition”. Higher average scores on the scale were indicative of greater self-compassion (Cronbach α = 0.85).

#### 2.2.2. Work Engagement and Exhaustion

The 16-item Oldenburg burnout inventory (OLBI) [5,6], which consists of two scales, was used to measure work engagement and exhaustion. Using a four-point Likert-type scale (1—strongly disagree; 4—strongly agree), participants were asked to indicate the level of agreement with each statement in relation to their work. Sample items are: “I find my work to be a positive challenge” (engagement; Cronbach α = 0.70) and “After work, I tend to need more time than in the past to relax and feel better” (exhaustion; Cronbach α = 0.81). Higher average scores on each scale were indicative of greater work engagement and exhaustion, respectively.

#### 2.2.3. Professional Life Satisfaction

The five-item satisfaction with life scale (SWLS) [15] was used to assess overall satisfaction with professional life. A minor modification was employed for this study, with the word “professional” added in each item (i.e., professional life) to prompt participants to think specifically about their professional life rather than life globally when responding to items. Using a seven-point Likert-type scale (1—strongly disagree; 7—strongly agree), participants were asked to indicate the degree of agreement with each statement in relation to their professional life. Sample items are: “In most ways, my professional life is close to my ideal” and “If I could restart my professional life, I would change almost nothing” (Cronbach α = 0.93). Higher average scores on the scale were indicative of greater satisfaction with professional life.

### 2.3. Analyses

SPSS 24.0 (IBM Corp., Armonk, NY, USA) was used to analyze the data (to be available at the institutional research data repository www.library.ualberta.ca/research-support/data-management). First, correlations were computed to determine if the associations among the study variables were in the expected direction. Next, sequential regression analyses [21] were performed to test the hypothesized relationships (Figure 1) and to examine the extent to which the relationship between self-compassion and professional life satisfaction was mediated by work engagement and exhaustion. Specifically, the first set of analyses regressed the proposed mediating variables of work engagement and exhaustion on self-compassion; the second set of analyses directly regressed professional life satisfaction on self-compassion; and the third set of analyses simultaneously regressed professional life satisfaction on self-compassion, engagement, and exhaustion. 

## 3. Results

Descriptive statistics for the study variables are shown in Table 1. All bivariate correlations among the variables in this study were moderately strong and significant (Table 1).

**Figure 2:**
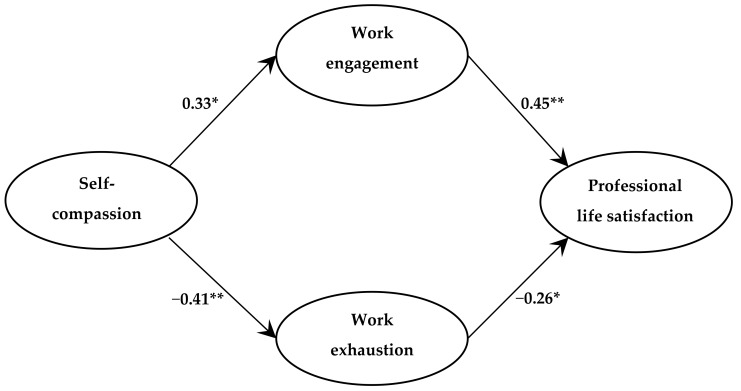
Summary of sequential regression analyses; ** *p* < 0.01; * *p* < 0.05.

The first set of regression analyses indicated that when work engagement and work exhaustion were regressed on self-compassion, self-compassion positively predicted work engagement (β = 0.33; *p* < 0.05) and negatively predicted work exhaustion (β = −0.41; *p* < 0.01). This confirmed the link between the predictor and mediating variables. The results of the second set of regression analyses (Table 2, Model 1), which examined the direct association between self-compassion and professional life satisfaction, indicated that self-compassion was positively associated with professional life satisfaction (β = 0.32; *p* < 0.05). The third set of regression analyses simultaneously regressed professional life satisfaction on self-compassion, work engagement, and work exhaustion (Table 2, Model 2). The direct association between self-compassion and professional life satisfaction reduced to the point of non-significance (β = 0.06; *p* > 0.05), indicating that the relationship between self-compassion and professional life satisfaction was mediated by work engagement and work exhaustion (Table 2, Model 2). Collectively, self-compassion, work engagement, and work exhaustion explained almost 40% of the variance in professional life satisfaction among the physicians in this study. The links between the study variables established in sequential regression analyses and respective coefficients are shown in Figure 2. While the figure may appear to portray causal associations, it should be understood that no causality can be inferred due to the correlational nature of the data.

## 4. Discussion

Healthcare practice holds numerous challenges, many of which include elements of uncertainty (e.g., clinical ambiguity, close calls, and therapeutic setbacks) [1]. The results of this study indicate that physicians who approach the practice of medicine with a self-compassionate mindset experience better professional wellbeing. Specifically, physicians who were more self-compassionate experienced greater work engagement and felt less exhausted due to work demands. In turn, these physicians were more satisfied with their professional life than those physicians practicing less self-compassion in uncertain and challenging times. These findings are reassuring for potential intervention because poor work engagement and exhaustion are aspects of work-related burnout [5,6], which adversely affects healthcare providers worldwide [7].

Importantly, self-compassion is an acquired skill—promoting self-compassionate frames of mind through psychoeducation and training programs has been shown to have positive and lasting effects in various achievement settings (e.g., school, sport) and in daily life [10,11,22,23]. In sport, for example, self-compassion interventions with athletes have been successful in helping them manage concerns over mistakes, self-criticism, and rumination in times of setbacks and uncertainty, which stands to further enhance athletic performance and psychological wellbeing [22]. A potential criticism of self-compassion and its possible downsides are concerns that too much self-compassion might result in passivity and relaxed standards of quality. However, such concerns do not appear to have empirical support. On the contrary, there is evidence indicating that self-compassion psychologically benefits the self and others [10], by providing emotional resilience when faced with uncertainty and challenges. In learning contexts, for example, high self-compassion among students has been linked to better transition to university [23]. In medical education, those students who are more self-compassionate appear to experience greater engagement with their studies, whereas those students who are less self-compassionate feel more exhausted from their studies [24]. Compassion for oneself is also shown to ameliorate burnout and secondary traumatic stress in medical students and residents [25]. In a recent national survey of US pediatric residents, self-compassion was shown to be longitudinally associated with lower stress and burnout [14]. On behalf of the Pediatric Resident Burnout–Resilience Study Consortium, the authors concluded self-compassion to be a promising interventional target for trainees [14]. This past research, along with the findings of the present study, provides empirical evidence in support of fostering self-compassionate frames of mind and coping strategies in medical trainees and practitioners. 

Considering the high-stress, high-stakes nature of medical practice and the ubiquitous presence of uncertainty in it, self-compassion initiatives are warranted in helping medical trainees and practitioners learn to recognize the nature of uncertainty, understand its underlying mechanisms, and manage it adaptively.

## 5. Limitations

Several important limitations within this study need to be considered. First, we were unable to compute the response rate due to the variety of means we used to recruit physicians into the study (e.g., mailing lists, word of mouth), which provided no ability to establish the number of potential participants contacted. Second, although we did not undertake a formal validity study for the professional life satisfaction scale, the pattern of relationships between the constructs in this study (see correlations in Table 1) provides some evidence of validity. Third, this study used self-reported data, which may pose concerns around a possible social desirability bias in the survey responses. Additionally, motivations of those physicians who chose to participate in the study and those who, for whatever reasons, decided not to, remain unknown. Nevertheless, it is reassuring that those physicians who chose to participate in this study showed a spectrum of background characteristics (e.g., gender, career stage, medical specialty). Study participants also varied with respect to the degrees of self-compassion, work engagement, work exhaustion, and professional life satisfaction, yielding evidence against social desirability bias in the survey responses. Additionally, individual participant responses were kept confidential, which decreases the risk of social desirability bias. Finally, although the sample size in this study was small, the minimum requirement of at least ten observations per variable for regression analysis was met. Despite the small sample size, the observed effects (correlations and regression coefficients) were moderately strong.

## 6. Conclusions

The findings of this study indicate that self-compassion is beneficial for the professional wellbeing of practicing physicians. Self-compassionate physicians appear to be more engaged and feel less exhausted due to work demands and as such, are more satisfied with their professional life than those physicians who exhibit less compassion toward themselves in uncertain and challenging times. Future research on how to support practicing physicians and medical trainees in becoming more self-compassionate is warranted, with implications for healthcare practice and education. Additionally, research examining the relationship of physician self-compassion with patient outcomes is needed due to the association of burnout with empathy fatigue [26,27].

## Figures and Tables

**Table 1:** Descriptive statistics for the study variables.

Variables	M (SD; R)	Correlations		
		Self-Compassion	Work Engagement	Work Exhaustion
Self-compassion	3.39 (0.60; 1.83–4.42)			
Work engagement	2.86 (0.36; 1.63–3.63)	0.33 *		
Work exhaustion	2.50 (0.43; 1.75–3.50)	−0.41 **	−0.50 **	
Prof. life satisfaction	5.24 (1.24; 1.40–7.00)	0.32 *	0.60 **	−0.51 **

** *p* < 0.01; * *p* < 0.05; *n* = 57; M—mean; SD—standard deviation; R—range. Self-compassion was measured on a five-point scale; work engagement and work exhaustion were measured on a four-point scale; professional (prof.) life satisfaction was measured on a seven-point scale.

**Table 2:** Standardized regression coefficients for self-compassion and mediating variables of work engagement and work exhaustion predicting professional life satisfaction.

	Professional Life Satisfaction	
	Model 1	Model 2
Self-compassion	0.32 *	0.06
Work engagement	–	0.45 **
Work exhaustion	–	–0.26 *
*F*-value	6.18 *	12.82 **
Total adjusted *R*^2^	0.09	0.39

** *p* < 0.01; * *p* < 0.05; *n* = 57.
